# Supplementary material for: Increased Vocalization of Rats in Response to Ultrasonic Playback as a Sign of Hypervigilance Following Fear Conditioning
Source: Brain Sci. 2021 Jul 23;11(8):970. doi: 10.3390/brainsci11080970 (PMC8393681; doi:10.3390/brainsci11080970)
Supplement: Supplementary file 1 [file brainsci-11-00970-s001.zip › brainsci-1225624-supplementary.pdf]

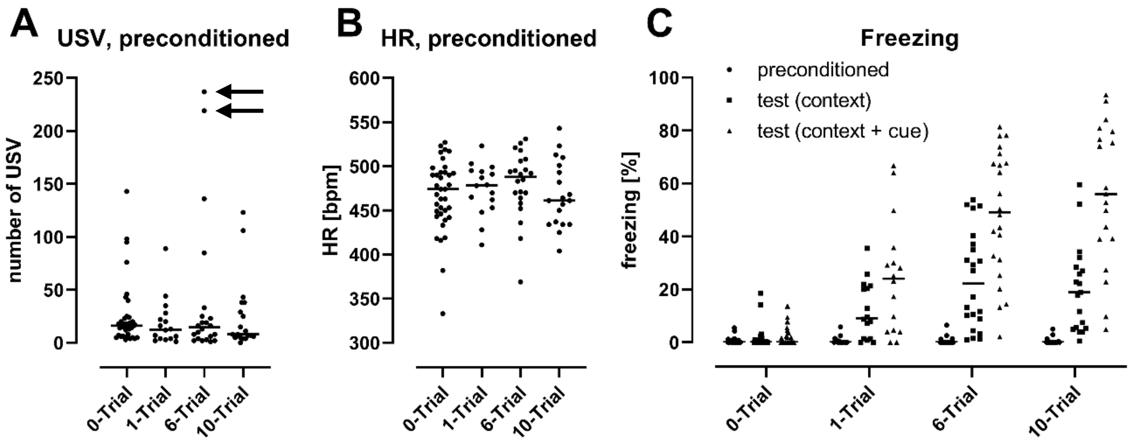

D

			Freezing		
**Group**	# of USV	HR	Pre (%)	Context (%)	Cue (%)
0-Trial, n = 37	25.2 ± 4.9	466.8 ± 6.7	0.4 ± 0.2	1.2 ± 0.6	0.5 ± 0.3
1-Trial, n = 16	19.2 ± 5.6	474.1 ± 7.3	0.7 ± 0.4	12.2 ± 2.7	23.9 ± 5.3
6-Trial, n = 20	21.6 ± 7.3	479.8 ± 8.1	0.6 ± 0.4	24.1 ± 4.3	38.4 ± 5.9
10-Trial, n = 19	25.3 ± 7.8	468.1 ± 8.6	0.5 ± 0.3	20.2 ± 3.8	55.7 ± 6.2
Group effect:	p = 0.5621	p = 0.4838	p = 0.7329	p = **0.0000**	p = **0.0000**

E

	Evaluation of differences in freezing							
	Feezing (cue)				Feezing (context)			
**Group**	Pre vs. Test	vs. 0-Trial	vs. 1-Trial	vs. 6-Trial	Pre vs. Test	vs. 0-Trial	vs. 1-Trial	vs. 6-Trial
0-Trial	0.6496	x	-		0.9096	x		
1-Trial	**0.0007**	**0.0000**	x		**0.0010**	**0.0000**	x	
6-Trial	**0.0001**	**0.0000**	0.1206	x	**0.0001**	**0.0000**	**0.0416**	x
10-Trial	**0.0001**	**0.0000**	**0.0010**	**0.0242**	**0.0001**	**0.0000**	0.1361	0.5779
Test used:	Wilcoxon	Mann-Whitney			Wilcoxon	Mann-Whitney		

Figure S1. Graph of emitted USV (**A**), heart rate (HR) (**B**) and freezing levels (**C**) of rats before conditioning (**A**, **B**, dots in **C**; with medians); and test freezing levels to context (5 min, squares) and context with cue (light; averaged three 20-s long exposures, triangles). Arrows in **A** show two copiously vocalizing rats which were not included in the final analysis. (**D**) Table of the values graphed and group effects (Kruskal-Willis). (**E**) Table of statistical comparisons of freezing between preconditional (pre) values and test values (Wilcoxon) as well as test values between groups (Mann-Whitney), with p < 0.05 values in bold.

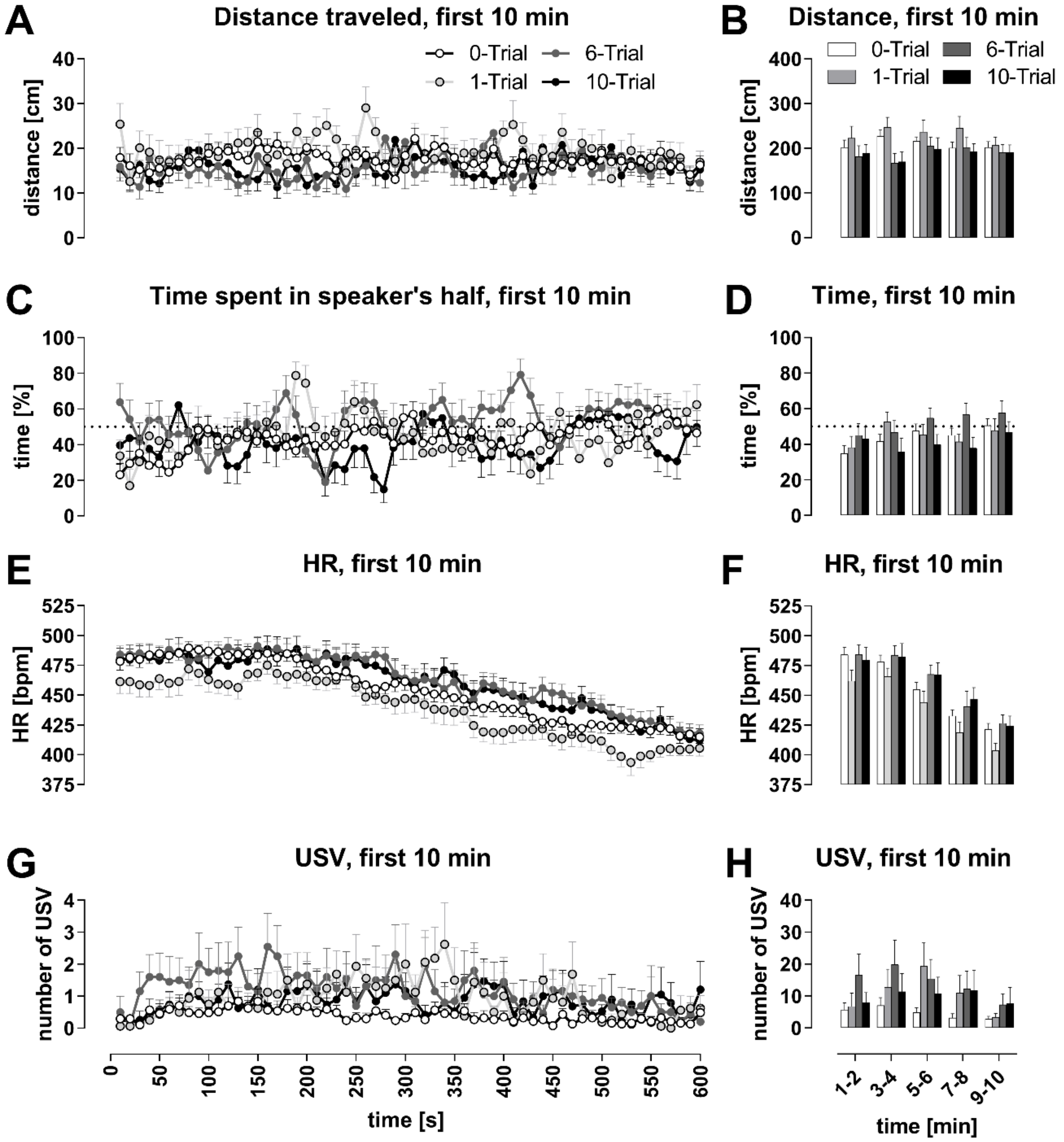

Figure S2. Assessment of locomotor activity, heart rate (HR) and the number of USV emitted of all animals during the first 10 min of silence in the experimental cage; (**A, B**) distance traveled (cm); (**C, D**) time (%) spent in a speaker's half of the cage with horizontal dotted line marking 50% chance level; results above it show more time in the speaker's half; (**E, F**) heart rate (bpm); (**G, H**) number of USV. Values are means with SEM. HR, initially stable, declined progressively in all four groups; however, HR in 1-Trial group was lower than in groups with zero, six and ten shocks. Mean values with SEM are presented for 10-s intervals in line graphs (**A**, **C**, **E**, **G**) and 2-min intervals in bar graphs (**B**, **D**, **F**, **H**); groups: 0-Trial, n = 37; 1-Trial, n = 16; 6-Trial, n = 20; 10-Trial, n = 19.

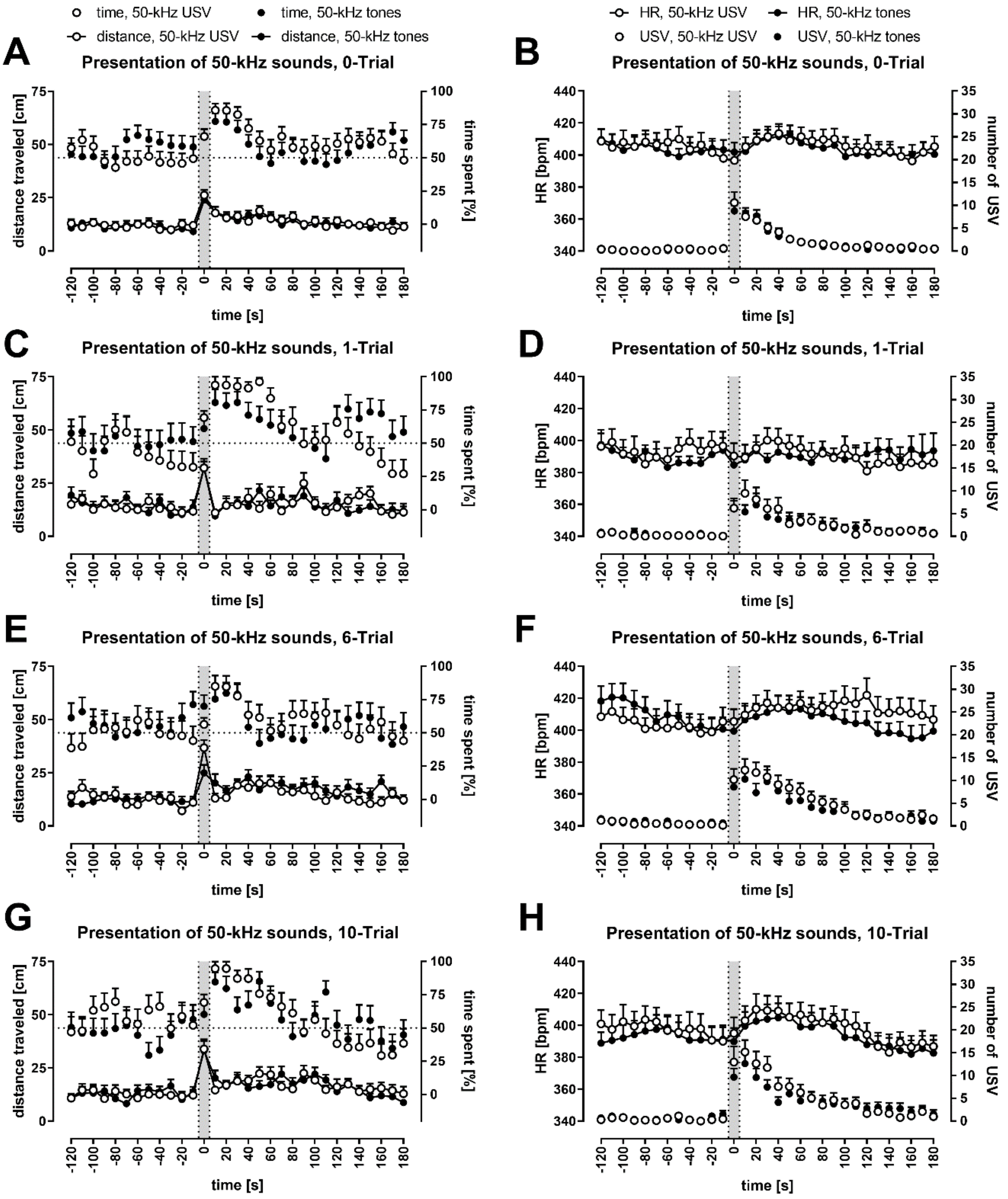

Figure S3. Effect of 50-kHz ultrasonic playback sounds on distance traveled, time spent in the speaker's half of the cage, heart rate (HR) and USV emission. Gray sections correspond to the 10-s-long ultrasonic presentation. Graphs depict responses after previous exposure to: no shock (**A**, **B**), one shock (**C**, **D**), six shocks (**E**, **F**), and ten shocks (**G**, **H**). In the left column (**A**, **C**, **E**, **G**), distance traveled is presented as connected dots (cm, left Y axis), percentage of time spent in the speaker's cage half – as not connected dots (%, right Y axis). In the right column (**B**, **D**, **F**, **H**), HR is presented as connected dots (bpm; beats per minute, left Y axis); the number of USV is presented as not connected dots (right Y axis). Each point is a mean for a 10-s-long time-interval with SEM. The dotted horizontal line marks a 50% chance value for time in a side of the cage. HR increased after playback in all groups except 1-Trial rats. There was also a clear USV response to the playback in all groups. The increase in locomotor activity to the playback was more pronounced in fear-conditioned (FC) animals than in 0-Trial rats; groups: 0-Trial, n = 37; 1-Trial, n = 16; 6-Trial, n = 20; 10-Trial, n = 19.

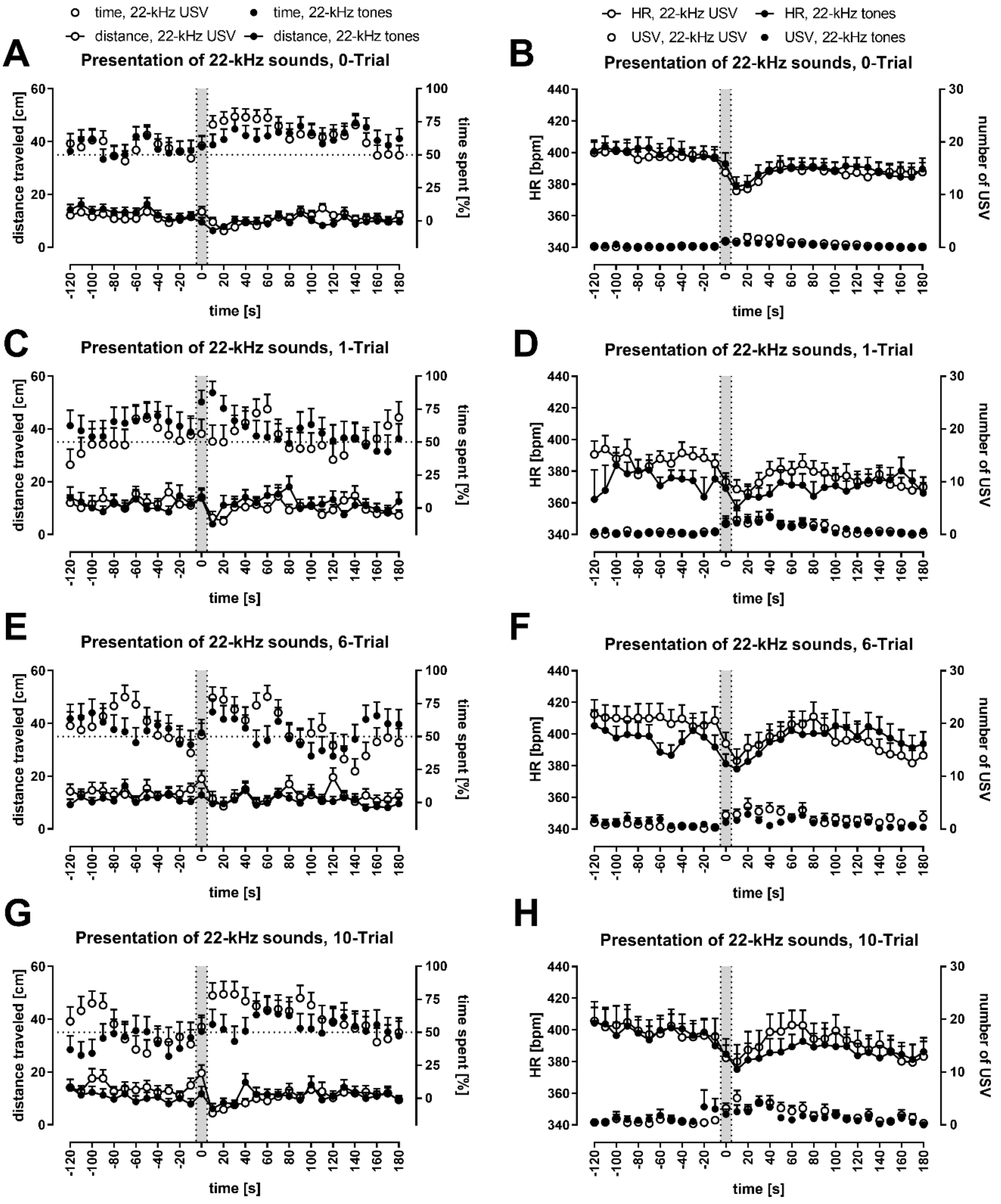

Figure S4. Effect of 22-kHz ultrasonic playback sounds on distance traveled, time spent in the speaker's half of the cage, heart rate (HR) and USV emission. Gray sections correspond to the 10-s-long ultrasonic presentation. Graphs depict responses after previous exposure to: no shock (**A**, **B**), one shock (**C**, **D**), six shocks (**E**, **F**), and ten shocks (**G**, **H**). In the left column (**A**, **C**, **E**, **G**), distance traveled is presented as connected dots (cm, left Y axis), percentage of time spent in the speaker's cage half – as not connected dots (%, right Y axis). In the right column (**B**, **D**, **F**, **H**), HR is presented as connected dots (bpm; beats per minute, left Y axis); the number of USV is presented as not connected dots (right Y axis). Each point is a mean for a 10-s-long time-interval with SEM. The dotted horizontal line marks a 50% chance value for time in a side of the cage. Playback exposure results in an HR drop in all four groups. There was also a clear USV response to the playback, especially in FC groups. The decrease in locomotor activity after the playback was more pronounced in FC animals than in 0-Trial rats; groups: 0-Trial, n = 37; 1-Trial, n = 16; 6-Trial, n = 20; 10-Trial, n = 19.

time-interval [s] / group	**0-Trial**	**1-Trial**	**6-Trial**	**10-Trial**	**all rats**	**0-Trial**	**1-Trial**	**6-Trial**	**10-Trial**	**all rats**
# of rats	n = 37	n = 16	n = 20	n = 19	n = 92	n = 37	n = 16	n = 20	n = 19	n = 92
**distance**										
	**50-kHz USV**					**22-kHz USV**				
-120 to 180	**0.0000**	**0.0049**	**0.0000**	**0.0001**	**0.0000**	**0.0012**	**0.0073**	0.5023	**0.0018**	**0.0000**
-60 to 60	**0.0000**	**0.0035**	**0.0000**	**0.0001**	**0.0000**	**0.0001**	**0.0010**	0.1131	**0.0002**	**0.0000**
-10 to 10	**0.0000**	**0.0001**	**0.0001**	**0.0007**	**0.0000**	0.1635	**0.0152**	**0.0150**	**0.0004**	**0.0000**
0 to 30	**0.0009**	**0.0000**	**0.0000**	**0.0134**	**0.0000**	**0.0001**	**0.0005**	**0.0037**	**0.0002**	**0.0000**
-120 to -100	0.3485	0.4724	0.7047	0.1426	0.7457	0.9733	0.7788	0.1572	0.6918	0.5153
-30 to -10	0.0855	0.6454	0.0907	0.2683	**0.0461**	0.1923	0.7788	0.3499	0.8539	0.6545
	**50-kHz tone**					**22-kHz tone**				
-120 to 180	**0.0000**	**0.0019**	**0.0001**	**0.0000**	**0.0000**	**0.0000**	0.1346	0.2252	**0.0943**	**0.0000**
-60 to 60	**0.0000**	**0.0012**	**0.0028**	**0.0007**	**0.0000**	**0.0004**	**0.0130**	0.1252	**0.0397**	**0.0000**
-10 to 10	**0.0000**	**0.0016**	**0.0260**	**0.0029**	**0.0000**	**0.0306**	**0.0053**	0.0863	**0.0120**	**0.0000**
0 to 30	**0.0172**	**0.0005**	0.0905	0.1657	**0.0000**	0.0846	**0.0023**	**0.0043**	**0.0207**	**0.0000**
-120 to -100	0.4820	0.1738	0.7047	0.6227	0.8404	0.7841	0.4437	0.5220	0.6918	0.7959
-30 to -10	0.9221	0.6456	1.0000	0.4308	0.9575	0.6489	0.4724	0.3867	0.6918	0.9892
	**50-kHz sounds**					**22-kHz sounds**				
-120 to 180	**0.0000**	**0.0002**	**0.0000**	**0.0000**	**0.0000**	**0.0000**	**0.0014**	0.0676	**0.0025**	**0.0000**
-60 to 60	**0.0000**	**0.0000**	**0.0000**	**0.0000**	**0.0000**	**0.0000**	**0.0004**	**0.0261**	**0.0014**	**0.0000**
-10 to 10	**0.0000**	**0.0002**	**0.0001**	**0.0002**	**0.0000**	**0.0306**	**0.0072**	0.0743	**0.0009**	**0.0000**
0 to 30	**0.0005**	**0.0000**	**0.0000**	**0.0010**	**0.0000**	**0.0017**	**0.0027**	**0.0072**	**0.0004**	**0.0000**
-120 to -100	0.4692	0.2691	0.5220	0.6918	0.8682	0.8276	0.6456	0.7047	0.5318	0.9267
-30 to -10	0.8276	0.4437	0.3867	0.2683	0.4237	**0.0275**	0.5698	0.5488	0.9487	0.2023

Table S1. Evaluation of changes in **distance traveled** around playback of ultrasounds (50-kHz USV, 50-kHz tones, 22-kHz USV, 22-kHz tones; with averaged results for USV and tones, i.e. sounds) presented during the 0 s time-interval (from -120 s to 180 s, -60 s to 60 s, -10 s to 10 s), as well as during the control intervals before playback (from -120 s to -100 s, -30 s to -10 s) observed in control rats (0-Trial), and rats which underwent fear-conditioning with different number of shocks (1-Trial, 6-Trial, 10-Trial) as well as all the rats analyzed together, all Friedman test, with $p < 0.05$ values in bold. Please note $p > 0.05$ values for -120 to -100 s and -30 to -10 s intervals.

**group:**	**0-Trial**	**1-Trial**	**6-Trial**	**10-Trial**	**all rats**	**0-Trial**	**1-Trial**	**6-Trial**	**10-Trial**	**all rats**
**# of rats**	n = 37	n = 16	n = 20	n = 19	n = 92	n = 37	n = 16	n = 20	n = 19	n = 92
**time in speaker's half vs. 50%**										
time-interval [s]	**50-kHz USV**					**22-kHz USV**				
0 s (playback)	**0.0089**	**0.0013**	0.1259	**0.0079**	**0.0000**	0.3694	0.6051	0.9851	0.6580	0.1173
10 s to 30 s	**0.0000**	**0.0023**	**0.0117**	**0.0002**	**0.0000**	**0.0009**	0.6233	**0.0090**	**0.0401**	**0.0000**
10 s to 60 s	**0.0001**	**0.0005**	**0.0333**	**0.0001**	**0.0000**	**0.0000**	0.2775	**0.0100**	0.0586	**0.0000**
-120 s to -100 s	0.2516	0.5695	0.4781	0.8405	0.7807	0.8268	0.5180	0.3507	0.1119	0.1279
-30 s to -10 s	0.7400	0.0980	0.7938	0.7172	0.3831	0.3694	0.7564	0.6274	0.7475	0.9101
	**50-kHz tone**					**22-kHz tone**				
0 s (playback)	**0.0115**	0.2343	**0.0383**	0.2954	**0.0001**	0.4644	**0.0174**	0.7369	0.8092	**0.0310**
10 s to 30 s	**0.0003**	**0.0038**	**0.0012**	**0.0048**	**0.0000**	0.0738	**0.0299**	0.1672	0.8721	**0.0058**
10 s to 60 s	**0.0123**	**0.0061**	**0.0366**	**0.0017**	**0.0000**	**0.0499**	0.0557	0.2180	0.6292	**0.0056**
-120 s to -100 s	0.9339	0.9588	0.3317	0.8092	0.7377	0.2078	0.4080	0.1730	0.0910	0.2949
-30 s to -10 s	0.2454	1.0000	0.2043	0.7782	0.1379	0.8504	0.2553	0.9702	0.3981	0.8993
	**50-kHz sounds**					**22-kHz sounds**				
0 s (playback)	**0.0004**	**0.0125**	**0.0249**	**0.0279**	**0.0000**	0.3219	**0.0294**	0.8753	0.7564	0.0531
10 s to 30 s	**0.0000**	**0.0007**	**0.0017**	**0.0003**	**0.0000**	**0.0003**	0.0830	**0.0072**	0.1005	**0.0000**
10 s to 60 s	**0.0002**	**0.0004**	**0.0206**	**0.0002**	**0.0000**	**0.0003**	**0.0438**	**0.0075**	0.0840	**0.0000**
-120 s to -100 s	0.3037	0.4899	0.6580	0.8261	0.4755	0.1102	0.9750	0.1788	0.7983	0.0810
-30 s to -10 s	0.4331	0.3343	0.6701	0.6192	0.6486	0.7613	0.3627	0.6603	0.4229	0.9534

Table S2. Evaluation **time spent** in the speaker's half values **in comparison with 50% chance level** during playback of ultrasounds, i.e. 50-kHz USV, 50-kHz tones, 22-kHz USV, 22-kHz tones, averaged USV and tones (sounds), i.e. during the 0 s time-interval, and immediately after (from 10 s to 30 s, 10 s to 60 s) as well as during the control intervals before playback (from -120 s to -100 s, -30 s to -10 s) observed in control rats (0-Trial), and rats which underwent fear-conditioning with different number of shocks (1-Trial, 6-Trial, 10-Trial) as well as all the rats analyzed together, all Wilcoxon test, with $p < 0.05$ values in bold. Please note $p > 0.05$ values for all -120 to -100 s and -30 to -10 s intervals.

time-interval [s] / group	**0-Trial**	**1-Trial**	**6-Trial**	**10-Trial**	**all rats**	**0-Trial**	**1-Trial**	**6-Trial**	**10-Trial**	**all rats**
# of rats	n = 37	n = 16	n = 20	n = 19	n = 92	n = 37	n = 16	n = 20	n = 19	n = 92
**time**										
	**50-kHz USV**					**22-kHz USV**				
-120 to 180	**0.0000**	**0.0000**	**0.0384**	**0.0000**	**0.0000**	**0.0000**	0.0790	**0.0000**	**0.0000**	**0.0000**
-60 to 60	**0.0000**	**0.0000**	**0.0034**	**0.0004**	**0.0000**	**0.0000**	0.1591	**0.0002**	**0.0000**	**0.0000**
-10 to 10	**0.0001**	**0.0001**	**0.0070**	**0.0007**	**0.0000**	**0.0012**	0.9623	**0.0023**	**0.0000**	**0.0000**
0 to 30	**0.0003**	**0.0001**	**0.0000**	**0.0012**	**0.0000**	**0.0017**	0.6957	**0.0035**	**0.0033**	**0.0000**
-120 to -100	0.5163	**0.0162**	0.2725	0.1629	0.8995	0.9520	0.1677	0.9608	0.0839	0.2259
-30 to -10	0.7903	0.8777	0.8700	0.6483	0.9931	0.1499	0.8465	0.3932	0.8777	0.2111
	**50-kHz tone**					**22-kHz tone**				
-120 to 180	**0.0021**	0.0930	0.0948	**0.0016**	**0.0000**	0.0719	**0.0492**	0.1855	0.3230	0.1396
-60 to 60	**0.0055**	0.0800	0.0628	**0.0001**	**0.0000**	0.0882	0.3111	0.3943	0.2053	**0.0028**
-10 to 10	0.1283	**0.0458**	0.4493	**0.0138**	**0.0000**	0.2927	**0.0098**	0.0762	0.3093	**0.0007**
0 to 30	**0.0407**	**0.0112**	0.6933	**0.0092**	**0.0000**	**0.0325**	0.1061	0.0803	0.7074	**0.0250**
-120 to -100	0.9810	0.6065	0.5890	0.9556	0.7463	0.7309	0.6188	0.5258	0.9661	0.8310
-30 to -10	0.9355	0.9592	0.0949	0.8574	0.6065	0.9200	0.8984	0.1188	**0.0366**	0.4939
	**50-kHz sounds**					**22-kHz sounds**				
-120 to 180	**0.0000**	**0.0000**	**0.0193**	**0.0000**	**0.0000**	**0.0000**	**0.0072**	**0.0000**	**0.0074**	**0.0000**
-60 to 60	**0.0000**	**0.0000**	**0.0012**	**0.0000**	**0.0000**	**0.0000**	0.7520	**0.0008**	**0.0041**	**0.0000**
-10 to 10	**0.0004**	**0.0005**	**0.0093**	**0.0008**	**0.0000**	**0.0005**	0.1869	**0.0002**	**0.0025**	**0.0000**
0 to 30	**0.0008**	**0.0011**	**0.0037**	**0.0008**	**0.0000**	**0.0006**	0.9224	**0.0006**	**0.0047**	**0.0000**
-120 to -100	0.6286	0.0522	0.5097	0.6361	0.6023	0.4766	0.8975	0.8357	0.5669	0.4390
-30 to -10	0.7221	0.7463	**0.0414**	0.4700	0.1639	0.2775	0.4124	0.1637	0.1870	0.2750

Table S3. Evaluation of changes in **time spent** in the speaker's half **around playback of ultrasounds** (50-kHz USV, 50-kHz tones, 22-kHz USV, 22-kHz tones; with averaged results for USV and tones, i.e. sounds) presented during the 0 s time-interval (from -120 s to 180 s, -60 s to 60 s, -10 s to 10 s), as well as during the control intervals before playback (from -120 s to -100 s, -30 s to -10 s) observed in control rats (0-Trial), and rats which underwent fear-conditioning with different number of shocks (1-Trial, 6-Trial, 10-Trial) as well as all the rats analyzed together, all Friedman test, with $p < 0.05$ values in bold. Please note $p > 0.05$ values for -120 to -100 s and -30 to -10 s intervals.

**group:**	**0-Trial**	**1-Trial**	**6-Trial**	**10-Trial**	**all rats**	**0-Trial**	**1-Trial**	**6-Trial**	**10-Trial**	**all rats**
**n**	n = 37	n = 16	n = 20	n = 19	n = 92	n = 37	n = 16	n = 20	n = 19	n = 92
**time in speaker's half**										
time-interval [s]	**50-kHz USV**					**22-kHz USV**				
before vs. playback	**0.0148**	**0.0031**	0.3760	**0.0356**	**0.0000**	0.2122	0.4413	0.6002	0.1520	0.0623
before vs. after	**0.0000**	**0.0059**	0.0557	**0.0027**	**0.0000**	**0.0058**	0.9594	**0.0171**	**0.0121**	**0.0000**
playback vs. after	**0.0046**	**0.0342**	**0.0065**	**0.0125**	**0.0000**	**0.0250**	0.7353	**0.0186**	**0.0051**	**0.0003**
	**50-kHz tone**					**22-kHz tone**				
before vs. playback	0.2414	0.4631	0.1579	0.3003	**0.0356**	0.5098	0.0663	0.6784	0.0929	**0.0239**
before vs. after	0.0938	**0.0277**	0.1627	0.1089	**0.0009**	0.0534	0.1386	0.1361	0.2604	**0.0051**
playback vs. after	0.1908	**0.0192**	0.7333	0.1337	**0.0054**	0.0926	0.4990	0.1549	1.0000	0.1315
	**50-kHz sounds**					**22-kHz sounds**				
before vs. playback	**0.0390**	**0.0084**	0.1365	**0.0347**	**0.0000**	0.2301	0.0745	0.5695	**0.0413**	**0.0079**
before vs. after	**0.0010**	**0.0025**	**0.0279**	**0.0042**	**0.0000**	**0.0018**	0.5076	**0.0139**	**0.0413**	**0.0000**
playback vs. after	**0.0072**	**0.0076**	**0.0126**	**0.0131**	**0.0000**	**0.0094**	0.7794	**0.0042**	**0.0342**	**0.0001**

Table S4. Evaluation of changes in time spent in the speaker's half **before** (i.e. average time spent from -30 s to -10 s time-intervals) vs. during **playback** (i.e. 0 s time-interval) vs. **after** the playback (i.e. average from 10 s to 30 s time-intervals); significant values ($p < 0.05$) marked in bold, all Wilcoxon test. Analyzed were the control rats (0-Trial), and rats which underwent fear-conditioning with different number of shocks (1-Trial, 6-Trial, 10-Trial) as well as all the rats together, which one day later were exposed to ultrasonic playbacks (50-kHz USV, 50-kHz tones, 22-kHz USV, 22-kHz tones; with averaged results for USV and tones, i.e. sounds). Please note more instances of significance in case of "sounds" in comparison with USV and tone playbacks.

time-interval [s] / group	**0-Trial**	**1-Trial**	**6-Trial**	**10-Trial**	**all rats**	**0-Trial**	**1-Trial**	**6-Trial**	**10-Trial**	**all rats**
# of rats	n = 37	n = 16	n = 20	n = 19	n = 92	n = 37	n = 16	n = 20	n = 19	n = 92
**HR changes**										
	**50-kHz USV**					**22-kHz USV**				
-120 to 180	**0.0000**	0.1092	**0.0244**	**0.0009**	**0.0000**	**0.0000**	**0.0001**	**0.0000**	**0.0015**	**0.0000**
-60 to 60	**0.0000**	0.1797	**0.0047**	**0.0235**	**0.0000**	**0.0000**	**0.0020**	**0.0004**	**0.0007**	**0.0000**
-10 to 10	0.0572	0.3798	0.4620	0.1956	0.1030	**0.0000**	0.0548	**0.0023**	0.0799	**0.0000**
0 to 30	**0.0000**	**0.0009**	0.2371	0.0998	**0.0000**	**0.0007**	0.1491	0.3470	0.2203	**0.0002**
-120 to -100	0.0974	0.3453	0.7736	0.5157	0.6169	0.7656	0.5506	0.9874	0.5203	0.8449
-30 to -10	0.2880	0.6514	0.6106	0.6918	0.5452	0.6547	0.4724	0.6065	0.4317	0.7644
	**50-kHz tone**					**22-kHz tone**				
-120 to 180	0.0834	0.8709	**0.0000**	**0.0180**	**0.0000**	**0.0000**	**0.0000**	**0.0026**	**0.0000**	**0.0000**
-60 to 60	**0.0316**	0.2341	0.0728	0.2995	**0.0000**	**0.0000**	**0.0000**	**0.0000**	**0.0017**	**0.0000**
-10 to 10	0.7613	0.2092	0.6376	0.2924	0.5068	**0.0004**	**0.0498**	**0.0032**	0.6850	**0.0000**
0 to 30	0.3566	**0.0244**	0.1444	0.2703	**0.0011**	**0.0023**	0.2490	0.0664	0.4954	**0.0001**
-120 to -100	0.3299	0.7396	0.5803	0.7762	0.5115	0.9529	0.3022	0.2972	0.1233	0.4441
-30 to -10	0.6259	0.8290	0.9120	0.8056	0.6967	0.4032	0.8628	0.2123	0.1105	0.0797
	**50-kHz sounds**					**22-kHz sounds**				
-120 to 180	**0.0001**	0.7374	**0.0000**	**0.0000**	**0.0000**	**0.0000**	**0.0000**	**0.0000**	**0.0000**	**0.0000**
-60 to 60	**0.0002**	0.3155	**0.0041**	**0.0227**	**0.0000**	**0.0000**	**0.0000**	**0.0000**	**0.0001**	**0.0000**
-10 to 10	0.1563	0.5698	0.1102	0.1335	**0.0146**	**0.0001**	**0.0033**	**0.0011**	0.2058	**0.0000**
0 to 30	**0.0006**	**0.0021**	0.0771	**0.0117**	**0.0000**	**0.0012**	0.1035	0.0640	0.2564	**0.0000**
-120 to -100	0.2500	0.8932	0.7287	0.4385	0.9645	0.9221	0.3679	0.3035	0.6918	0.9442
-30 to -10	0.5289	0.6319	0.9512	0.9487	0.8737	0.5843	0.6457	0.8607	0.0979	0.3004

Table S5. Evaluation of changes in **heart rate (HR)** around playback of ultrasounds (50-kHz USV, 50-kHz tones, 22-kHz USV, 22-kHz tones; with averaged results for USV and tones, i.e. sounds) presented during the 0 s time-interval (from -120 s to 180 s, -60 s to 60 s, -10 s to 10 s), as well as during the control intervals before playback (from -120 s to -100 s, -30 s to -10 s) observed in control rats (0-Trial), and rats which underwent fear-conditioning with different number of shocks (1-Trial, 6-Trial, 10-Trial) as well as all the rats analyzed together, all Friedman test, with $p < 0.05$ values in bold. Please note $p > 0.05$ values for all -120 to -100 s and -30 to -10 s intervals.

**group:**	**0-Trial**	**1-Trial**	**6-Trial**	**10-Trial**	**all rats**	**0-Trial**	**1-Trial**	**6-Trial**	**10-Trial**	**all rats**
**n**	n = 37	n = 16	n = 20	n = 19	n = 92	n = 37	n = 16	n = 20	n = 19	n = 92
**heart rate (HR)**										
time-interval [s]	**50-kHz USV**					**22-kHz USV**				
before vs. playback	0.0750	0.1183	0.5135	0.7628	0.2406	**0.0007**	**0.0386**	**0.0057**	**0.0269**	**0.0000**
before vs. 10 s	0.4417	0.2775	0.2180	0.2514	0.2008	**0.0001**	**0.0038**	**0.0111**	**0.0074**	**0.0000**
before vs. 10-30 s	**0.0475**	0.8160	0.0894	**0.0429**	**0.0027**	**0.0000**	**0.0027**	**0.0366**	0.0582	**0.0000**
before vs. 10-60 s	**0.0190**	0.8767	**0.0479**	**0.0298**	**0.0003**	**0.0004**	**0.0494**	0.0930	0.5595	**0.0000**
-10 s vs. playback	0.7186	0.1406	0.5595	0.3341	0.9494	**0.0059**	0.0783	**0.0007**	**0.0298**	**0.0000**
-10 s vs. 10 s	0.0797	0.2013	0.5016	0.0702	0.0607	**0.0001**	**0.0140**	**0.0064**	**0.0166**	**0.0000**
playback vs. 10 s	**0.0047**	0.7564	0.4898	0.1531	**0.0117**	**0.0089**	0.3259	0.1454	0.8092	**0.0031**
playback vs. 10-30 s	**0.0004**	0.3942	0.0967	0.0510	**0.0000**	**0.0052**	0.4380	0.6274	0.7022	**0.0318**
playback vs. 10-60 s	**0.0001**	0.2553	0.0826	0.0702	**0.0000**	0.1184	1.0000	0.6580	0.1842	0.9795
	**50-kHz tone**					**22-kHz tone**				
before vs. playback	0.9932	0.0703	0.7938	0.7782	0.3971	0.8327	0.2443	**0.0012**	**0.0382**	**0.0027**
before vs. 10 s	0.8741	0.7761	0.5016	0.1474	0.3012	**0.0002**	**0.0106**	**0.0005**	**0.0242**	**0.0000**
before vs. 10-30 s	0.1818	0.7564	0.2514	0.1221	**0.0187**	**0.0000**	**0.0174**	**0.0008**	0.1313	**0.0000**
before vs. 10-60 s	0.0733	0.7764	**0.0366**	0.1213	**0.0020**	**0.0011**	0.0627	**.0313***	0.1978	**0.0000**
-10 s vs. playback	0.7933	0.1118	0.9553	0.8617	0.5176	0.8741	0.1550	**0.0106**	0.5595	**0.0338**
-10 s vs. 10 s	0.9000	0.6909	0.4666	0.1474	0.4965	**0.0002**	**0.0019**	**0.0021**	0.1119	**0.0000**
playback vs. 10 s	0.8505	0.3011	0.2959	0.1387	0.1408	**0.0002**	0.0736	0.3411	0.0976	**0.0000**
playback vs. 10-30 s	0.1006	0.1148	0.0702	0.1262	**0.0016**	**0.0076**	0.1406	0.7369	0.5197	**0.0118**
playback vs. 10-60 s	**0.0128**	0.0666	**0.0187**	0.1262	**0.0001**	0.8327	0.4851	**0.0124**	0.7475	0.6375
	**50-kHz sounds**					**22-kHz sounds**				
before vs. playback	0.3231	0.0591	0.7089	0.8563	0.2266	0.0660	0.0787	**0.0004**	**0.0166**	**0.0000**
before vs. 10 s	0.7514	0.2220	0.1913	0.1165	0.2343	**0.0000**	**0.0019**	**0.0027**	**0.0141**	**0.0000**
before vs. 10-30 s	0.0535	0.8767	0.0793	**0.0329**	**0.0022**	**0.0000**	**0.0023**	**0.0046**	0.0586	**0.0000**
before vs. 10-60 s	**0.0304**	0.9588	**0.0169**	**0.0242**	**0.0002**	**0.0001**	**0.0174**	**0.0366**	0.1842	**0.0000**
-10 s vs. playback	0.8628	0.0832	0.5135	0.6791	0.6551	0.1597	**0.0299**	**0.0006**	**0.0475**	**0.0000**
-10 s vs. 10 s	0.3832	0.3011	0.3165	0.0872	0.1587	**0.0000**	**0.0031**	**0.0007**	**0.0294**	**0.0000**
playback vs. 10 s	0.1239	0.8160	0.1305	0.0684	**0.0062**	**0.0011**	0.0879	0.0872	0.3760	**0.0001**
playback vs. 10-30 s	**0.0018**	0.1337	**0.0124**	**0.0329**	**0.0000**	**0.0040**	0.1550	0.6274	0.9679	**0.0077**
playback vs. 10-60 s	**0.0004**	0.0557	**0.0036**	**0.0329**	**0.0000**	0.0635	0.6051	0.1474	0.3341	0.7470

Table S6. Evaluation of changes in heart rate (HR) around the time of ultrasonic playback, i.e. between **before** (i.e. average time spent from -30 s to -10 s time-intervals) or **immediately before** (at -10 s time-interval) vs. during **playback** (i.e. 0 s time –interval) vs. **after** the playback (i.e. at 10 s time-interval, vs. average from 10 s to 30 s or from 10 s to 60 s time-intervals); significant values ($p < 0.05$) marked in bold, all Wilcoxon test. Analyzed were the control rats (0-Trial), and rats which underwent fear-conditioning with different number of shocks (1-Trial, 6-Trial, 10-Trial) as well as all the rats together, which one day later were exposed to ultrasonic playbacks (50-kHz USV, 50-kHz tones, 22-kHz USV, 22-kHz tones; with averaged results for USV and tones, i.e. sounds). Please note the prevalence of significant values in the group of all rats; * – not significant when two outlying rats were included.

**Differences in heart rate (HR) values following 50-kHz vs. 22-kHz playback**															
**time**	**50-kHz USV vs. 22-kHz USV**					**50-kHz tones vs. 22-kHz tones**					**50-kHz sounds vs. 22-kHz sounds**				
groups	**0-Trial**	**1-Trial**	**6-Trial**	**10-Trl.**	**all rats**	**0-Trial**	**1-Trial**	**6-Trial**	**10-Trl.**	**all rats**	**0-Trial**	**1-Trial**	**6-Trial**	**10-Trl.**	**all rats**
**-120**	0.4924	0.9176	0.5067	0.6580	0.9322	0.1065	0.0703	0.3803	0.0910	0.1121	0.1971	0.2775	0.5379	0.2684	0.3198
**-110**	0.9219	0.6233	0.8789	0.7782	0.9423	0.6151	0.1706	0.1403	0.1446	0.3116	0.7514	0.2343	0.3604	0.3958	0.4286
**-100**	0.5923	0.8767	0.7172	0.8092	0.9023	0.6887	0.5695	0.1615	0.2684	0.4873	0.4699	0.7960	0.4330	0.4939	0.5607
**-30**	0.4110	0.5180	0.9359	0.7939	0.4914	0.5362	0.1089	0.8248	0.6435	0.4824	0.2363	0.2343	0.8083	0.6165	0.3937
**-20**	0.7860	0.3259	0.9839	0.5328	0.8184	0.1843	0.0525	0.8519	0.6165	0.2057	0.1843	**0.0386**	0.7089	0.4094	0.2922
**-10**	0.8268	0.2553	0.8373	0.6726	0.8274	0.3515	0.1406	0.6951	0.9839	0.1225	0.4783	0.0929	0.7228	0.8405	0.2515
**0**	0.1353	0.0744	0.0793	0.2684	**0.0021**	0.1080	0.1272	0.0731	0.5732	**0.0064**	0.0882	**0.0465**	**0.0276**	0.0949	**0.0002**
**10**	**0.0001**	0.0980	**0.0160**	**0.0186**	**0.0000**	**0.0002**	**0.0008**	**0.0124**	**0.0294**	**0.0000**	**0.0000**	**0.0106**	**0.0080**	**0.0249**	**0.0000**
**20**	**0.0000**	**0.0090**	**0.0363**	0.0559	**0.0000**	**0.0000**	**0.0048**	**0.0152**	0.1119	**0.0000**	**0.0000**	**0.0016**	**0.0080**	0.0534	**0.0000**
**30**	**0.0004**	**0.0174**	**0.0038**	0.0559	**0.0000**	**0.0006**	**0.0072**	**0.0290**	**0.0347**	**0.0000**	**0.0002**	**0.0052**	**0.0051**	**0.0269**	**0.0000**
**40**	**0.0005**	0.0627	**0.0400**	0.3760	**0.0000**	**0.0004**	**0.0016**	0.0570	0.0836	**0.0000**	**0.0004**	**0.0131**	**0.0008**	0.1712	**0.0000**
**50**	**0.0071**	0.0832	0.0582	0.7172	**0.0011**	**0.0002**	0.0664	0.1851	**0.0346**	**0.0000**	**0.0002**	0.0703	**0.0479**	0.2049	**0.0000**
**60**	**0.0069**	0.1089	0.1084	0.9039	**0.0014**	**0.0103**	**0.0309**	0.1354	0.1650	**0.0001**	**0.0007**	**0.0262**	0.1084	0.4813	**0.0000**
**70**	**0.0038**	0.3520	0.2250	0.8721	**0.0068**	**0.0143**	0.0664	0.3507	0.2049	**0.0010**	**0.0008**	0.2243	0.3048	0.3838	**0.0003**
**80**	**0.0079**	0.1477	0.5016	0.5732	**0.0055**	0.0696	**0.0004**	0.3604	0.1071	**0.0002**	**0.0045**	**0.0072**	0.3317	0.2352	**0.0001**
**90**	**0.0005**	0.0787	0.1560	0.4460	**0.0002**	**0.0085**	**0.0186**	0.3905	0.3760	**0.0006**	**0.0003**	**0.0174**	0.2043	0.3547	**0.0000**
**100**	**0.0008**	**0.0262**	**0.0303**	0.9199	**0.0001**	0.0855	0.0557	0.8519	0.7172	**0.0357**	**0.0026**	**0.0140**	0.1354	0.7782	**0.0004**
**110**	**0.0027**	0.1406	0.0894	0.5732	**0.0004**	0.1115	**0.0083**	0.8519	0.4688	**0.0161**	**0.0045**	**0.0200**	0.3317	0.4939	**0.0006**
**120**	**0.0012**	0.2775	0.0894	0.8721	**0.0012**	0.0955	**0.0229**	0.4222	0.7771	**0.0087**	**0.0030**	0.1272	0.1169	0.5869	**0.0005**
**130**	**0.0056**	0.1148	0.1506	0.8248	**0.0025**	0.0750	**0.0362**	0.7228	0.4326	**0.0343**	**0.0047**	0.0703	0.5135	0.8092	**0.0038**
**140**	**0.0201**	**0.0468**	**.0438***	0.7628	**0.0020**	**0.0169**	0.1089	0.9839	0.6009	**0.0088**	**0.0039**	**0.0437**	0.2396	0.7782	**0.0007**
**150**	0.1049	0.0879	**0.0152**	0.9679	**0.0015**	**0.0177**	0.1406	0.8228	0.8789	**0.0277**	**0.0210**	0.0929	0.1560	0.8405	**0.0025**
**160**	0.2032	**0.0105**	**0.0349**	0.5197	**0.0034**	**0.0050**	0.0664	0.8373	0.9679	**0.0102**	**0.0069**	**0.0319**	0.2043	0.6292	**0.0009**
**170**	**0.0499**	**0.0214**	**0.0251**	0.1474	**0.0001**	**0.0006**	0.0832	0.6677	0.4688	**0.0006**	**0.0016**	**0.0299**	0.1005	0.2954	**0.0000**
**180**	0.0814	0.1089	0.0522	0.7172	**0.0033**	**0.0424**	0.0557	0.6813	0.2954	**.0464***	**0.0202**	**0.0200**	0.1506	0.8721	**0.0012**

Table S7. Statistical evaluation of comparisons between the effects of **50-kHz vs. 22-kHz playback** of USV, tones, as well as of USV and tones averaged (sounds) presented during 0 s time-intervals **on heart rate (HR)** – as reported for time-intervals between -120 s and 180 s; significant values ($p < 0.05$) marked in bold, all Wilcoxon test. Analyzed were the control rats (0-Trial), and rats which underwent fear-conditioning the day before – with different number of shocks (1-Trial, 6-Trial, 10-Trial), as well as all the rats together. Please note the onset of significant values to coincide with the time of the playbacks; * – not significant when two outlying rats were included.

	50-kHz USV-playback					50-kHz tones-playback					50-kHz sounds-playback				
**groups:**	**0-T**	**6-T**	**10-T**	**6-10-T**	**other**	**0-T**	**6-T**	**10-T**	**6-10-T**	**other**	**0-T**	**6-T**	**10-T**	**6-10-T**	**other**
**HR changes**	**vs. 1-Trial rats**					**vs. 1-Trial rats**					**vs. 1-Trial rats**				
before vs. 10 s	0.1909	0.0885	0.1046	0.0569	0.0799	0.6281	0.5037	0.2397	0.2950	0.4011	0.3137	0.1519	0.0792	0.0693	0.1223
-10 vs. 10 s	**0.0485**	0.1810	0.0547	0.0638	**0.0371**	0.6989	0.4639	0.1496	0.2177	0.3698	0.2185	0.1225	0.0507	**0.0456**	0.0781
before vs. 10-30 s	0.2334	0.1114	0.0821	0.0569	0.0911	0.4556	0.5665	0.2968	0.3589	0.3646	0.1909	0.1660	**0.0314**	**0.0453**	0.0704
-10 vs. 10-30 s	0.0532	0.1864	0.0608	0.0693	**0.0411**	0.5788	0.3385	0.1798	0.1914	0.3044	0.1211	0.1346	**0.0240**	**0.0329**	**0.0444**
before vs. 10-60 s	0.1601	**0.0484**	0.0851	**0.0345**	0.0541	0.2570	0.2934	0.2083	0.1882	0.1822	0.1211	0.0561	**0.0314**	**0.0199**	**0.0343**
-10 vs. 10-60 s	**0.0254**	0.0746	0.0637	**0.0379**	**0.0186**	0.4335	0.2934	0.1277	0.1432	0.2167	0.0551	0.0667	**0.0341**	**0.0233**	**0.0221**

Table S8. Comparisons of **heart rate (HR) changes, i.e. differences in HR value between two periods, in 1-Trial group** (the rats received one electric shock the day before) **vs. other groups** (control, 0-Trial/0-T group; the rats which received 6 shocks, 6-Trial/6-T; 10 shocks, 10-Trial/10-T; the 6-Trial and 10-Trial rats combined, 6-10-T, and all non-1-Trial rats combined, other); significant values ($p < 0.05$) marked in bold, all Mann-Whitney test.

time-interval [s] / group	**0-Trial**	**1-Trial**	**6-Trial**	**10-Trial**	**all rats**	**0-Trial**	**1-Trial**	**6-Trial**	**10-Trial**	**all rats**
# of rats	n = 37	n = 16	n = 20	n = 19	n = 92	n = 37	n = 16	n = 20	n = 19	n = 92
**number of USV emitted**										
	**50-kHz USV**					**22-kHz USV**				
-120 to 180	**0.0000**	**0.0000**	**0.0000**	**0.0000**	**0.0000**	**0.0000**	**0.0001**	**0.0000**	**0.0000**	**0.0000**
-60 to 60	**0.0000**	**0.0000**	**0.0000**	**0.0000**	**0.0000**	**0.0000**	**0.0106**	**0.0000**	**0.0000**	**0.0000**
-10 to 10	**0.0000**	**0.0002**	**0.0001**	0.4967	**0.0000**	**0.0073**	0.2111	**0.0084**	0.3130	**0.0000**
0 to 30	**0.0000**	0.2299	0.8482	**0.0001**	**0.0011**	0.9963	0.5558	0.9420	**0.0150**	0.8622
-120 to -100	0.1869	0.5404	0.9078	0.1409	0.4755	0.3902	0.8669	0.2927	0.6918	0.2883
-30 to -10	0.0701	0.2319	0.6065	0.3679	0.1528	0.4459	0.1462	0.1738	0.1615	0.8348
	**50-kHz tone**					**22-kHz tone**				
-120 to 180	**0.0000**	**0.0000**	**0.0000**	**0.0000**	**0.0000**	**0.0001**	**0.0005**	**0.0318**	**0.0000**	**0.0000**
-60 to 60	**0.0000**	**0.0000**	**0.0000**	**0.0000**	**0.0000**	**0.0025**	**0.0042**	0.0642	**0.0000**	**0.0000**
-10 to 10	**0.0000**	**0.0001**	**0.0001**	0.1942	**0.0000**	**0.0454**	0.8007	0.1778	0.0675	**0.0044**
0 to 30	**0.0001**	0.2660	0.1779	**0.0001**	**0.0000**	0.9558	0.3345	0.6676	0.3679	0.1597
-120 to -100	0.2276	0.2122	0.2276	0.5308	0.1738	0.3583	0.2574	0.2090	0.9394	0.8765
-30 to -10	**0.0067**	0.4937	0.8382	**0.0441**	0.1975	0.7047	0.1653	0.1561	0.8071	0.3189
	**50-kHz sounds**					**22-kHz sounds**				
-120 to 180	**0.0000**	**0.0000**	**0.0000**	**0.0000**	**0.0000**	**0.0000**	**0.0000**	**0.0000**	**0.0000**	**0.0000**
-60 to 60	**0.0000**	**0.0000**	**0.0000**	**0.0000**	**0.0000**	**0.0000**	**0.0001**	**0.0000**	**0.0000**	**0.0000**
-10 to 10	**0.0000**	**0.0000**	**0.0001**	**0.0266**	**0.0000**	**0.0065**	0.1128	**0.0012**	0.1346	**0.0000**
0 to 30	**0.0000**	0.0705	0.4621	**0.0000**	**0.0000**	0.9974	0.7748	0.7665	0.0963	0.4743
-120 to -100	0.0921	0.2167	0.7491	0.0558	**0.0291**	0.7463	0.4046	0.1620	0.7548	0.8233
-30 to -10	**0.0030**	0.1466	0.8623	**0.0320**	0.1597	0.9775	0.2801	1.0000	0.2101	0.4878

Table S9. Evaluation of changes in the **number of USV** emitted **around playback** of ultrasounds (50-kHz USV, 50-kHz tones, 22-kHz USV, 22-kHz tones; with averaged results for USV and tones, i.e. sounds) presented during the 0 s time-interval (from -120 s to 180 s, -60 s to 60 s, -10 s to 10 s), as well as during the control intervals before playback (from -120 s to -100 s, -30 s to -10 s) observed in control rats (0-Trial), and rats which underwent fear-conditioning with different number of shocks (1-Trial, 6-Trial, 10-Trial) as well as all the rats analyzed together, all Friedman test, with $p < 0.05$ values in bold. Please note $p > 0.05$ values for -120 to -100 s and -30 to -10 s intervals.

**group:**	**0-Trial**	**1-Trial**	**6-Trial**	**10-Trial**	**all rats**	**0-Trial**	**1-Trial**	**6-Trial**	**10-Trial**	**all rats**
**n**	n = 37	n = 16	n = 20	n = 19	n = 92	n = 37	n = 16	n = 20	n = 19	n = 92
**number of USV emitted**										
time-interval [s]	**50-kHz USV**					**22-kHz USV**				
before vs. playback	**0.0000**	**0.0027**	**0.0005**	**0.0003**	**0.0000**	**0.0168**	0.2367	**0.0060**	**0.0077**	**0.0000**
before vs. 10 s	**0.0000**	**0.0022**	**0.0008**	**0.0005**	**0.0000**	**0.0107**	0.2249	**0.0209**	**0.0029**	**0.0000**
before vs. 10-30 s	**0.0000**	**0.0015**	**0.0008**	**0.0004**	**0.0000**	**0.0025**	**0.0357**	**0.0022**	**0.0022**	**0.0000**
before vs. 10-120 s	**0.0000**	**0.0010**	**0.0004**	**0.0005**	**0.0000**	**0.0006**	**0.0093**	**0.0004**	**0.0004**	**0.0000**
	**50-kHz tone**					**22-kHz tone**				
before vs. playback	**0.0000**	**0.0019**	**0.0015**	**0.0002**	**0.0000**	**0.0284**	0.1159	0.3980	0.3105	**0.0024**
before vs. 10 s	**0.0000**	**0.0012**	**0.0015**	**0.0002**	**0.0000**	**0.0058**	0.1159	0.0972	0.5541	**0.0007**
before vs. 10-30 s	**0.0000**	**0.0014**	**0.0004**	**0.0002**	**0.0000**	**0.0035**	**0.0277**	**0.0099**	0.0712	**0.0000**
before vs. 10-120 s	**0.0000**	**0.0018**	**0.0002**	**0.0003**	**0.0000**	**0.0014**	**0.0044**	**0.0045**	0.1330	**0.0000**
	**50-kHz sounds**					**22-kHz sounds**				
before vs. playback	**0.0000**	**0.0008**	**0.0004**	**0.0002**	**0.0000**	**0.0137**	0.1235	**0.0157**	**0.0342**	**0.0000**
before vs. 10 s	**0.0000**	**0.0008**	**0.0005**	**0.0001**	**0.0000**	**0.0014**	0.1159	**0.0037**	**0.0108**	**0.0000**
before vs. 10-30 s	**0.0000**	**0.0008**	**0.0003**	**0.0002**	**0.0000**	**0.0012**	**0.0173**	**0.0018**	**0.0052**	**0.0000**
before vs. 10-120 s	**0.0000**	**0.0006**	**0.0002**	**0.0003**	**0.0000**	**0.0002**	**0.0037**	**0.0004**	**0.0229**	**0.0000**

Table S10. Evaluation of changes in the **number of USV** around the time of ultrasonic playback, i.e. between **before** (i.e. average time spent from -30 s to -10 s time-intervals) vs. during **playback** (i.e. 0 s time –interval) vs. **after** the playback (i.e. at 10 s time-interval, vs. average from 10 s to 30 s or from 10 s to 120 s time-intervals); significant values ($p < 0.05$) marked in bold, all Wilcoxon test. Analyzed were the control rats (0-Trial), and rats which underwent fear-conditioning with different number of electric shocks (1-Trial, 6-Trial, 10-Trial) as well as all the rats together, which one day later were exposed to ultrasonic playbacks (50-kHz USV, 50-kHz tones, 22-kHz USV, 22-kHz tones; with averaged results for USV and tones, i.e. sounds). Please note the prevalence of significant values in all the groups.

Differences in the number of USV following 50-kHz vs. 22-kHz playback															
**time**	**50-kHz USV vs. 22-kHz USV**					**50-kHz tones vs. 22-kHz tones**					**50-kHz sounds vs. 22-kHz sounds**				
groups	**0-Trial**	**1-Trial**	**6-Trial**	**10-Trl.**	**all rats**	**0-Trial**	**1-Trial**	**6-Trial**	**10-Trl.**	**all rats**	**0-Trial**	**1-Trial**	**6-Trial**	**10-Trl.**	**all rats**
-120	0.5698	**0.0431**	0.6566	0.6858	0.4427	0.8734	0.3452	0.6744	0.7263	0.9904	0.5360	0.0587	0.9063	0.8658	0.3298
-110	0.1599	0.0935	0.6726	0.3433	0.0661	0.8582	0.8551	0.6002	0.4755	0.7364	0.3454	0.4412	0.6356	0.3305	0.2070
-100	0.5098	0.3613	0.9645	0.3452	0.5098	0.6114	0.2851	0.7998	1.0000	0.4456	0.3233	0.0935	0.5761	0.4148	0.2257
-30	0.7089	0.1088	0.9326	0.1088	0.7938	0.6496	0.9326	NR	0.3452	0.6496	0.4072	0.0679	0.5541	0.1730	0.7775
-20	0.4859	0.2733	0.5930	0.5002	0.6359	0.7089	0.5930	0.7150	0.4772	0.9032	0.9569	0.5541	0.7150	0.7989	0.9569
-10	0.7282	NR	0.5294	0.5002	0.7282	0.9184	0.5294	0.1380	0.8886	0.8570	0.9937	0.1056	0.6744	0.7671	0.7996
0	**0.0000**	0.0597	**0.0052**	**0.0065**	**0.0000**	**0.0000**	**0.0231**	**0.0019**	**0.0007**	**0.0000**	**0.0000**	**0.0202**	**0.0010**	**0.0009**	**0.0000**
10	**0.0000**	**0.0164**	**0.0008**	**0.0050**	**0.0000**	**0.0000**	**0.0277**	**0.0015**	**0.0003**	**0.0000**	**0.0000**	**0.0092**	**0.0007**	**0.0010**	**0.0000**
20	**0.0000**	**0.0207**	**0.0018**	**0.0050**	**0.0000**	**0.0000**	0.1551	**0.0033**	**0.0006**	**0.0000**	**0.0000**	**0.0360**	**0.0006**	**0.0008**	**0.0000**
30	**0.0000**	0.1141	**0.0015**	**0.0121**	**0.0000**	**0.0000**	0.1549	**0.0021**	0.0979	**0.0000**	**0.0000**	**0.0342**	**0.0007**	**0.0139**	**0.0000**
40	**0.0000**	0.3863	**0.0106**	0.1055	**0.0001**	**0.0000**	0.8127	**0.0015**	0.3152	**0.0000**	**0.0000**	0.3824	**0.0016**	0.1075	**0.0000**
50	**0.0011**	1.0000	**0.0052**	0.0557	**0.0027**	**0.0000**	0.2300	**0.0135**	**0.0086**	**0.0000**	**0.0000**	0.7007	**0.0009**	**0.0121**	**0.0000**
60	**0.0005**	0.3505	**0.0048**	0.0787	**0.0003**	**0.0000**	0.1551	**0.0120**	**0.0042**	**0.0000**	**0.0000**	0.3454	**0.0012**	**0.0060**	**0.0000**
70	**0.0055**	0.5076	**0.0288**	0.2209	**0.0128**	**0.0020**	0.4990	0.1330	0.1467	**0.0050**	**0.0006**	0.5937	**0.0229**	0.0664	**0.0017**
80	**0.0009**	0.6726	**0.0150**	0.0935	**0.0022**	**0.0006**	0.2135	0.1159	**0.0254**	**0.0016**	**0.0002**	0.3863	**0.0184**	**0.0199**	**0.0006**
90	**0.0056**	0.7353	**0.0113**	0.2945	**0.0132**	**0.0022**	0.3454	0.3078	**0.0454**	**0.0048**	**0.0007**	0.6566	**0.0186**	**0.0382**	**0.0017**
100	**0.0263**	0.5286	0.1261	0.1361	0.0587	**0.0001**	0.0910	0.0593	**0.0499**	**0.0001**	**0.0001**	0.2860	**0.0310**	**0.0258**	**0.0002**
110	**0.0080**	0.5930	0.2845	0.0506	**0.0169**	**0.0002**	0.5937	0.1851	**0.0185**	**0.0003**	**0.0001**	0.5049	0.1005	**0.0119**	**0.0001**
120	**0.0100**	0.0910	0.4769	0.4755	**0.0180**	**0.0019**	0.3270	0.2411	**0.0180**	**0.0033**	**0.0005**	0.1235	0.3454	**0.0150**	**0.0012**
130	0.1110	0.5286	0.7671	0.6465	0.1696	**0.0346**	0.8339	0.7794	0.1688	**0.0473**	**0.0154**	0.7598	0.8385	0.2393	**0.0305**
140	**0.0280**	0.8927	0.2604	0.1823	**0.0339**	**0.0000**	NR	**0.0117**	**0.0117**	**0.0000**	**0.0001**	0.7874	**0.0454**	**0.0076**	**0.0001**
150	0.4545	0.6002	0.4069	0.9326	0.6959	**0.0012**	1.0000	**0.0425**	0.0663	**0.0021**	**0.0143**	0.5408	0.2026	0.1029	**0.0360**
160	0.0536	0.2249	0.4412	0.5076	0.0761	**0.0007**	**0.0431**	**0.0251**	0.0687	**0.0004**	**0.0001**	0.0910	**0.0077**	0.4420	**0.0001**
170	**0.0125**	0.1088	0.1235	0.5940	**0.0174**	0.0799	NR	0.2626	0.2076	**.0323***	**0.0030**	0.1088	**.0284***	0.2049	**0.0011**
180	0.0953	0.1088	0.9057	0.1775	0.0953	0.0520	1.0000	0.1730	**0.0431**	**.0324***	**0.0249**	0.1441	0.6241	0.0910	**0.0180**

Table S11. Statistical evaluation of comparisons between the effects of **50-kHz vs. 22-kHz playback** of USV, tones, as well as of USV and tones averaged (sounds) presented during 0 s time-intervals **on the number of emitted vocalizations (USV)** – as reported for time-intervals between -120 s and 180 s; significant values ($p < 0.05$) marked in bold, all Wilcoxon test. Analyzed were the control rats (0-Trial), and rats which underwent fear-conditioning the day before – with different number of shocks (1-Trial, 6-Trial, 10-Trial), as well as all the rats together. Please note the onset of significant values to coincide with the onset of the playbacks; NR – the test gave no results; * – not significant when two outlying rats were included.

groups	number of USV				parameters of 50-kHz USV		
USV	total USV	50-kHz USV	short 22-kHz USV	long 22-kHz USV	duration [ms]	n	MPF [kHz]
**USV emitted to 50-kHz USV-playback (0–120 s time-intervals)**							
0-Trial	41.2 ± 7.1	39.9 ± 6.9	0.9 ± 0.2	0.3 ± 0.3	27.0 ± 1.7	19	61.7 ± 2.2
1-Trial	59.1 ± 19.5	57.8 ± 19.3	1.3 ± 0.6	0.0 ± 0.0	26.9 ± 2.1	9	59.7 ± 1.6
6-Trial	86.4 ± 16.5	85.7 ± 16.5	0.8 ± 0.3	0.0 ± 0.0	29.3 ± 2.6	14	61.1 ± 0.6
10-Trial	97.5 ± 17.5	95.8 ± 17.2	1.7 ± 0.7	0.0 ± 0.0	30.1 ± 1.8	15	60.8 ± 0.9
all FC'ed	82.3 ± 10.3	81.0 ± 10.2	1.2 ± 0.3	0.0 ± 0.0	29.1 ± 1.3	38	60.7 ± 0.6
all rats	65.7 ± 7.1	64.5 ± 7.0	1.1 ± 0.2	0.1 ± 0.1	28.4 ± 1.0	57	61.0 ± 0.8
**USV emitted to 22-kHz USV-playback (0–120 s time-intervals)**							
0-Trial	13.5 ± 3.6 ###	12.9 ± 3.6 ###	0.6 ± 0.3	0.0 ± 0.0	21.9 ± 2.1		58.1 ± 1.9
1-Trial	14.5 ± 5.4 ##	13.4 ± 5.3 ##	1.1 ± 0.6	0.0 ± 0.0	20.2 ± 2.4 #		54.5 ± 3.9 #
6-Trial	32.2 ± 7.6 ###	31.9 ± 7.6 ###	0.3 ± 0.1	0.0 ± 0.0	29.4 ± 2.4		60.1 ± 0.7
10-Trial	30.9 ± 9.8 ##	29.9 ± 9.8 ##	1.1 ± 0.5	0.0 ± 0.0	23.8 ± 2.4 #		58.9 ± 1.8
all FC'ed	26.6 ± 4.7 ###	25.8 ± 4.7 ###	0.8 ± 0.3	0.0 ± 0.0	25.1 ± 1.5 ##		58.4 ± 1.1 ##
all rats	21.4 ± 3.2 ###	20.6 ± 3.2 ###	0.7 ± 0.2	0.0 ± 0.0	24.0 ± 1.2 ###		58.3 ± 1.0 ##
**USV emitted to 50-kHz tone-playback (0–120 s time-intervals)**							
0-Trial	41.4 ± 6.6	40.4 ± 6.5	1.0 ± 0.4	0.0 ± 0.0	27.0 ± 2.0	18	58.6 ± 0.9
1-Trial	49.3 ± 16.2	48.6 ± 16.1	0.8 ± 0.2	0.0 ± 0.0	23.8 ± 2.9	9	58.2 ± 1.8
6-Trial	76.0 ± 18.5	74.6 ± 18.4	1.4 ± 0.5	0.0 ± 0.0	32.8 ± 3.3	11	61.0 ± 0.6
10-Trial	76.3 ± 15.3	75.4 ± 15.2	0.9 ± 0.4	0.0 ± 0.0	31.0 ± 2.1	13	61.1 ± 0.5
all FC'ed	68.3 ± 9.7	67.3 ± 9.7	1.0 ± 0.2	0.0 ± 0.0	29.6 ± 1.7	33	60.3 ± 0.6
all rats	57.5 ± 6.5	56.5 ± 6.5	1.0 ± 0.2	0.0 ± 0.0	28.7 ± 1.3	51	59.7 ± 0.5
**USV emitted to 22-kHz tone-playback (0–120 s time-intervals)**							
0-Trial	6.6 ± 2.1 ###	6.1 ± 2.0 ###	0.5 ± 0.3	0.0 ± 0.0	18.2 ± 2.1 #		55.5 ± 2.0
1-Trial	16.0 ± 6.9 #	15.3 ± 6.9 #	0.7 ± 0.5	0.0 ± 0.0	28.5 ± 7.9		54.2 ± 3.0
6-Trial	18.1 ± 5.5 ###	17.8 ± 5.5 ###	0.4 ± 0.2	0.0 ± 0.0	28.7 ± 3.0		59.4 ± 0.6
10-Trial	23.3 ± 8.1 ###	23.2 ± 8.1 ###	0.1 ± 0.1 #	0.0 ± 0.0	25.1 ± 2.2 ##		58.3 ± 1.4
all FC'ed	19.3 ± 3.9 ###	18.9 ± 3.9 ###	0.4 ± 0.2 ##	0.0 ± 0.0	27.2 ± 2.5 ##		57.5 ± 1.0 #
all rats	14.2 ± 2.6 ###	13.8 ± 2.6 ###	0.4 ± 0.1 ##	0.0 ± 0.0	24.0 ± 1.8 ###		56.8 ± 1.0 ##
**USV emitted to 50-kHz sound-playback (0–120 s time-intervals)**							
0-Trial	41.3 ± 6.0	40.1 ± 5.9	1.0 ± 0.3	0.1 ± 0.1	27.7 ± 1.5	24	60.7 ± 1.1
1-Trial	54.2 ± 17.4	53.2 ± 17.2	1.0 ± 0.4	0.0 ± 0.0	24.0 ± 2.1	12	59.6 ± 1.2
6-Trial	81.2 ± 16.4	80.1 ± 16.3	1.1 ± 0.3	0.0 ± 0.0	29.8 ± 2.8	14	60.4 ± 0.6
10-Trial	86.9 ± 15.1	85.6 ± 15.0	1.3 ± 0.4	0.0 ± 0.0	29.2 ± 1.8	16	60.9 ± 0.7
all FC'ed	75.3 ± 9.4	74.2 ± 9.3	1.1 ± 0.2	0.0 ± 0.0	27.9 ± 1.3	42	60.4 ± 0.5
all rats	61.6 ± 6.3	60.5 ± 6.3	1.1 ± 0.2	0.1 ± 0.1	27.8 ± 1.0	66	60.5 ± 0.5
**USV emitted to 22-kHz sound-playback (0–120 s time-intervals)**							
0-Trial	10.1 ± 2.7 ###	9.5 ± 2.6 ###	0.6 ± 0.3	0.0 ± 0.0	18.4 ± 1.6 ##		58.3 ± 1.6
1-Trial	15.3 ± 4.7 #	14.3 ± 4.7 #	0.9 ± 0.4	0.0 ± 0.0	27.8 ± 6.8		51.9 ± 3.4 ##
6-Trial	25.2 ± 5.9 ###	24.8 ± 5.9 ###	0.3 ± 0.1 #	0.0 ± 0.0	28.3 ± 2.1		59.8 ± 0.6
10-Trial	27.1 ± 8.3 ###	26.6 ± 8.3 ###	0.6 ± 0.3	0.0 ± 0.0	23.2 ± 2.2 ##		58.0 ± 1.6 #
all FC'ed	23.0 ± 3.8 ###	22.4 ± 3.8 ###	0.6 ± 0.2 #	0.0 ± 0.0	26.1 ± 2.0 ##		57.1 ± 1.2 ###
all rats	17.8 ± 2.6 ###	17.2 ± 2.6 ###	0.6 ± 0.1 ##	0.0 ± 0.0	23.2 ± 1.5 ###		57.6 ± 0.9 ###

Table S12. Comparison of **number of USV of different types** and **selected characteristics of 50-kHz USV** emitted during playback sessions, i.e. during the 10-s-long playback and 110 s afterwards, **in response to 50- vs. 22-kHz playback** (USV, tone and sounds) in control (0-Trial, n = 37), fear-conditioned (1-Trial, n = 16; 6-Trial, n = 20; 10-Trial, n = 19; all fear-conditioned/FC'ed), and all rats. MPF – mean peak frequency; categories of USV: 50-kHz (MPF >32 kHz), short 22-kHz (MPF of 18–32 kHz, duration <0.3 s), long 22-kHz (18–32 kHz, >0.3 s); significance levels are given for corresponding 50- vs. 22-kHz sounds, i.e. # p < 0.05, ## p < 0.01, ### p < 0. 001, all Wilcoxon test. Please note, duration and MPF of 50-kHz USV were compared only in rats which emitted USV in response to both 50- and 22-kHz playback, n of animals are given. A single USV was eliminated as a long outlier (with > 6 x SD). USV emitted in reaction to 22-kHz playbacks were less numerous, shorter and of lower MPF.

**groups**	**number of USV**				**parameters of 50-kHz USV**		
USV	**total USV**	**50-kHz USV**	**short 22-kHz USV**	**long 22-kHz USV**	**duration [ms]**	**n**	**MPF [kHz]**
**USV emitted to 50-kHz USV-playback (0–120 s time-intervals)**							
0-Trial	41.2 ± 7.1	39.9 ± 6.9	0.9 ± 0.2	0.3 ± 0.3	26.6 ± 1.6	30	60.6 ± 1.6
1-Trial	59.1 ± 19.5	57.8 ± 19.3	1.3 ± 0.6	0.0 ± 0.0	24.8 ± 2.2	15	60.4 ± 1.1
6-Trial	86.4 ± 16.5	85.7 ± 16.5	0.8 ± 0.3	0.0 ± 0.0	29.5 ± 2.4	15	61.2 ± 0.6
10-Trial	97.5 ± 17.5	95.8 ± 17.2	1.7 ± 0.7	0.0 ± 0.0	30.6 ± 1.8	19	60.9 ± 0.7
all FC'ed	82.3 ± 10.3	81.0 ± 10.2	1.2 ± 0.3	0.0 ± 0.0	28.5 ± 1.2	49	60.8 ± 0.5
all rats	65.7 ± 7.1	64.5 ± 7.0	1.1 ± 0.2	0.1 ± 0.1	27.8 ± 1.0	79	60.7 ± 0.7
**USV emitted to 50-kHz tone-playback (0–120 s time-intervals)**							
0-Trial	41.4 ± 6.6	40.4 ± 6.5	1.0 ± 0.4	0.0 ± 0.0	27.5 ± 1.4		58.8 ± 0.8*
1-Trial	49.3 ± 16.2	48.6 ± 16.1	0.8 ± 0.2	0.0 ± 0.0	25.4 ± 2.2		60.6 ± 1.4
6-Trial	76.0 ± 18.5	74.6 ± 18.4	1.4 ± 0.5	0.0 ± 0.0	30.1 ± 2.9		60.1 ± 1.1
10-Trial	76.3 ± 15.3	75.4 ± 15.2	0.9 ± 0.4	0.0 ± 0.0	31.7 ± 2.2		60.6 ± 0.6
all FC'ed	68.3 ± 9.7*	67.3 ± 9.7*	1.0 ± 0.2	0.0 ± 0.0	29.3 ± 1.4		60.5 ± 0.6
all rats	57.5 ± 6.5	56.5 ± 6.5	1.0 ± 0.2	0.0 ± 0.0	28.6 ± 1.0		59.8 ± 0.5*
**USV emitted to 22-kHz USV-playback (0–120 s time-intervals)**							
0-Trial	13.5 ± 3.6	12.9 ± 3.6	0.6 ± 0.3	0.0 ± 0.0	23.5 ± 2.2	16	56.9 ± 1.9
1-Trial	14.6 ± 5.4	13.4 ± 5.3	1.1 ± 0.6	0.0 ± 0.0	18.8 ± 3.1	8	57.5 ± 2.2
6-Trial	32.2 ± 7.6	31.9 ± 7.6	0.3 ± 0.1	0.0 ± 0.0	31.6 ± 2.7	11	60.1 ± 0.7
10-Trial	30.9 ± 9.8	29.9 ± 9.8	1.1 ± 0.5	0.0 ± 0.0	25.7 ± 2.4	12	61.0 ± 0.6
all FC'ed	26.6 ± 4.7	25.8 ± 4.7	0.8 ± 0.3	0.0 ± 0.0	26.0 ± 1.7	31	59.8 ± 0.7
all rats	21.4 ± 3.2	20.6 ± 3.2	0.7 ± 0.2	0.0 ± 0.0	25.2 ± 1.4	47	58.8 ± 0.8
**USV emitted to 22-kHz tone-playback (0–120 s time-intervals)**							
0-Trial	6.6 ± 2.1**	6.1 ± 2.0**	0.5 ± 0.3	0.0 ± 0.0	19.3 ± 2.2		54.0 ± 2.1*
1-Trial	16.0 ± 6.9	15.3 ± 6.9	0.7 ± 0.5	0.0 ± 0.0	19.7 ± 3.7		58.3 ± 1.8
6-Trial	18.1 ± 5.5	17.8 ± 5.5*	0.4 ± 0.2	0.0 ± 0.0	28.7 ± 3.0		59.4 ± 0.6
10-Trial	23.3 ± 8.1	23.2 ± 8.1	0.1 ± 0.1	0.0 ± 0.0	26.3 ± 2.0		57.9 ± 1.5
all FC'ed	19.3 ± 3.9	18.9 ± 3.9	0.4 ± 0.2	0.0 ± 0.0	25.4 ± 1.7		58.5 ± 0.8
all rats	14.2 ± 2.6**	13.8 ± 2.6**	0.4 ± 0.1	0.0 ± 0.0	23.3 ± 1.4		57.0 ± 0.9

Table S13. Comparison of **number of USV of different types** and **selected characteristics of 50-kHz USV** emitted during playback sessions, i.e. during the 10-s-long playback and 110 s afterwards, **in response to USV- vs. tone-playback** in control (0-Trial, n = 37), fear-conditioned (1-Trial, n = 16; 6-Trial, n = 20; 10-Trial, n = 19; all fear-conditioned, all FC'ed), and all rats. MPF – mean peak frequency; three categories of USV are: 50-kHz USV (MPF >32 kHz), short 22-kHz (MPF of 18–32 kHz, duration <0.3 s), long 22-kHz (MPF of 18–32 kHz, duration >0.3 s); significance levels are given for corresponding USV- vs. tone-playbacks, i.e. * $p < 0.05$, ** $p < 0.01$, all Wilcoxon test. Please note, the duration and MPF of 50-kHz USV were compared only in rats which emitted USV in response to both USV- and tone-playback, and relevant n of animals are given. USV emitted in reaction to tone playbacks turned out to be less numerous and of lower MPF.

**Differences in time spent in speaker's half following USV- vs. tone-playbacks**										
**group:**	**0-Trial**	**1-Trial**	**6-Trial**	**10-Trial**	**all rats**	**0-Trial**	**1-Trial**	**6-Trial**	**10-Trial**	**all rats**
**# of rats**	n = 37	n = 16	n = 20	n = 19	n = 92	n = 37	n = 16	n = 20	n = 19	n = 92
time-interval [s]	**50-kHz playback; USV vs tone**					**22-kHz playback; USV vs. tone**				
0 s (playback)	0.7275	0.5936	0.0949	0.4631	0.9258	0.9757	0.0926	0.7782	0.9250	0.4763
10 s to 30 s	0.1005	0.1386	0.2787	**0.0342**	**0.0020**	0.2418	0.1141	0.3343	0.0528	0.1779
10 s to 60 s	0.0670	**0.0231**	0.1701	0.0627	**0.0004**	0.1215	0.6949	0.1701	0.0692	**0.0236**
-120 s to -100 s	0.3505	0.4900	0.0555	0.6791	0.7790	0.7704	0.2721	0.4777	**.0309***	0.8076
-30 s to -10 s	0.2329	0.1730	0.1981	0.8971	0.0593	0.8824	0.6566	0.7939	0.6496	0.8710

Table S14. Differences in time spent in the speaker's half **in response to USV- vs. tone-playbacks** evaluated during playback (0 s time-interval), after playback (10 s to 30 s, 10 s to 60 s), as well as during the control intervals (from -120 s to -100 s, -30 s to -10 s), in control rats (0-Trial), and rats which underwent fear-conditioning with different number of shocks (1-Trial, 6-Trial, 10-Trial) as well as all the rats analyzed together, all Wilcoxon test, with p < 0.05 values in bold. Please note, USV-playback evoked more pronounced approach of the speaker, e.g. in “all rats” group; * – not significant when two outlying rats were included.
